# Thyroidectomy in the Treatment of Goitre

**Published:** 1895-03

**Authors:** John B. Roberts


### Selections and Abstracts.

### THYROIDECTOMY IN THE TREATMENT OF GOITRE.

By JOHN B. ROBERTS, M. D.

My object in bringing this topic before you is to call attention to the ease with which the enlarged thyroid gland can be enucleated and the mechanical symptoms caused by its pressure removed. After medicinal treatment has been employed sufficiently long to make it evident that no important change in size is to be expected, it seems to me that thyroidectomy need not be delayed.

Perhaps two months is as long as one need wait if, during that time the approved remedies have been employed in full doses. It must be remembered that goitres not infrequently vary in bulk, without relation to medical treatment; hence, a diminution is not necessarily the result of the local or internal medication. If dyspnœa, due to pressure on the trachea, is marked, or if hoarseness from compression of the recurrent laryngeal nerves is present, one month's unsuccessful treatment by medicines would probably induce me to operate.

Parenchymatous injections of alcohol, tincture of iodine, or other irritants, do not seem sufficiently successful, or free from danger, to be adopted as a routine, though cases may still come under my care in which I shall be willing to try them. Tapping or incision, and packing of cystic goitre, are available; but after the former, the cyst is liable to refill, and the latter operation makes almost as much scar as, and is not much less serious than, thyroidectomy. Electrolysis does not seem to have given sufficiently certain results to make it attractive. Tracheotomy may be demanded when tracheal compression is marked, though, in some cases, sub-cutaneous or open division of the deep cervical fascia to allow the bronchocele to bulge forward will avert the threatened suffocation.

Division of the thyroid isthmus to lessen pressure, or ligation of the thyroid arteries to cause atrophy, or prevent increased growth, are operations almost, if not quite, as serious as removal of a portion of the diseased gland.

I now speak only of thyroidectomy, or removal of a portion of the thyroid glands; total extirpation is unwarranted, because the occurrence of myxœdema as a result of the entire loss of the thyroid gland is pretty well established, experimentally and clinically. The most disfiguring and most compressing part of the goitre may be removed, and a portion of one lobe left to carry on the function of the gland. I would do this, even if the part left was apparently not healthy to the naked eye. It seems pretty well established that a small portion of the gland, or a small accessory gland, if such be present, is sufficient to avert the occurrence of myxœdema in patients subjected to removal of goitrous masses.

Thyroid feeding, the administration of thyroid extract, or the implantation of the thyroid gland of one of the lower animals, in the connective tissue or the peritoneal cavity of patients previously subjected to extirpation of the gland, may overcome the tendency to myxœdema; but, until this is really proved, the surgeon should do only a thyroidectomy, and leave a portion of the gland in position.

I shall not go over the steps of the operation in detail, as they are sufficiently clear to any operator who is prepared to deal with sharp hemorrhage, and who is careful enough to avoid injuring the recurrent laryngeal nerves. A vertical incision is made in the median line of the neck, or above the most prominent part of the tumor, and the necessary portion of the fibroid, cystic, or hypertrophic gland enucleated. Perfect asepsis, or antisepsis, is essential.

The enlarged thyroid in exophthalmic goitre has been subjected to operation, but the general character of this affection would deter me from operating upon the bronchocele, which is only one symptom of the disease; unless further statistics show its value in an unmistakable manner.

I add the reports of two cases, to illustrate the subject. Neither goitre was of great size, but both were annoying to the patients. They were radically cured by operation. The enormous fibroid goitres, seen especially in Europe, would, of course, be more difficult to deal with, especially in regard to the control of bleeding.

THYROIDECTOMY FOR GOITRE OF EIGHT YEARS' DURATION. RECOVERY.

A single woman, aged twenty-one years, with a family history of phthisis, first noticed enlargement of the thyroid gland when she began to menstruate, at the age of thirteen. The enlargement rapidly increased for three years, when it began to press upon the trachea to such an extent that respiration was interfered with, and she was obliged to give up going to school because of the dyspnœa that occurred while walking. After the lapse of a year, the bronchocele began to diminish in size, but, for the past three or four years, had not changed much in bulk.

The patient had been under medical treatment for some time for the thyroid enlargement, without diminution in the growth; and I, therefore, determined in April, 1891, to excise the most prominent portion of the gland. A four-inch incision was accordingly made in the median line of the neck, over the most prominent portion of the enlargement. The middle lobe or isthmus of the gland was first enucleated, and I subsequently removed the entire right lobe. In the removal, the sheath of the common carotid artery, with the descending branch of the ninth nerve upon it, was clearly exposed to view. A portion of the left lobe of the gland was then enucleated, making the size of the mass removed about that of a closed fist. The arteries requiring ligation were quite large and numerous. Sublimate gauze dressing was applied with considerable pressure, in order to avoid oozing.

About half an hour after the operation, the patient became so cyanotic from the pressure that the dressing, which had be come saturated with blood, was removed. Her pulse became rapid and weak, but the removal of the dressing, the application of hot water, the administration of digitalis, and other restoratives, were followed by cessation of bleeding and quick reaction. A clean dressing was immediately applied. The next day the patient was in good condition, with normal temperature. The wound healed promptly, without suppuration or marked swelling. The patient was sent to the sea-shore about three weeks after the operation, with the wound healed, though her voice was quite husky. This was probably due to interference at the time of operation with the recurrent laryngeal nerve.

The woman has been seen repeatedly since operation, the last time being nearly three years from the date of the thyroidectomy. Her health has continued good; her voice has recovered its normal quality, and there is no evidence whatever of myxœdema. It was because of the fear that myxœdema might occur that I allowed a portion of the left lobe of the enlarged gland to remain. The goitre seemed to be of the ordinary hypertrophic variety, though no microscopic examination was made. The contour of the neck is almost perfect, except for the unimportant scar; and there has been no further growth in the portion of the gland allowed to remain.

THYROIDECTOMY FOR CYSTIC GOITRE OF TWO YEARS' DURATION RECOVERY.

A young man, aged seventeen, had noticed two years previously a small swelling in the front of his throat about an inch and a half to the right and some distance below the Adam's apple. The tumor was not painful, but steadily increased in size until at the time he came under my observation there was some interference with respiration. He came to Philadelphia for the purpose of having the growth removed a year ago, but the surgeon who examined him advised against operative interference. When I first saw him, in November, 1892, the patient was the subject of a well marked goitre. The girth of his neck over the most prominent part of the tumor was 15¾ inches. The enlargement involved both lobes of the thyroid gland, but the right was larger than the left. Stridulous respiration was marked, even when he was perfectly quiet and not taking any exercise. Breathing became easier as soon as the growth on the right side was pushed away from the trachea. This phenomenon I repeatedly tested, and it was very marked. Above the main mass on each side was a small nodule, somewhat separated from the rest of the tumor, but evidently being the upper portion of the lobe on the corresponding side. There was a well marked groove between the two lobes, showing little involvement of the isthmus, and a furrow between the upper nodule and the main mass on each side. There was no exophthalmus, but there existed a doubtful systolic murmur.

I treated the patient for some weeks with digitalis and quinine, with ointment of the red iodide of mercury and lanoline externally, and subsequently with fluid extract of ergot.

After a month's treatment I determined to remove the prominent portion of the gland, which was making pressure upon the trachea. The patient took ether rather badly, as was to be expected from the interference with the respiration due to the growth. The right lobe of the tumor was exposed and was found to be cystic. The cyst was opened, and about an ounce and a half of dark-brown fluid was evacuated. The small nodule above and the right lobe containing the cyst were dissected from the under lying structures. The sheath of the carotid artery was exposed, and the lower portion of the mass extended close to the sheath of the subclavian artery.

Stridor was apparent after the operation; and some dyspnœa remained, which was, however, relieved to a considerable extent by loosening the bandage. Four days later the dressing was removed. Union by first intention seemed to have taken place. A dressing of gauze and collodion was applied, and on the sixth day after operation the patient was allowed to get up. Four days later a slight hemorrhage occurred through the gauze and collodion dressing, as the result of a fit of coughing. Slight oozing took place for a couple of weeks through the opening made by the giving way of the union at this time. The blood-clot contained in the cavity left by the excision of the right lobe of the gland did not break down into pus, but gradually became organized. The patient's temperature was normal during nearly the whole of the convalescence, though at one time shortly after the operation it rose to 101°. The patient was discharged about five weeks after the operation. His breathing had become normal, and the appearance of his neck was greatly improved, though there was still, of course, enlargement of the left lobe. There was no hoarseness remaining as the result of the operation, as in the former case.

A year later the patient wrote to me that he had had no trouble in breathing, that his voice was normal, and that there was no change in the appearance of his neck. The operation, therefore, was in every way satisfactory.

DISCUSSION.

DR. G. A. MUEHLECK said: I have a case under observation at present with a well-marked hypertrophic goitre which I am treating after the methcd of Gare, which is practised at Prof. Bruns' clinic at Tubingen. It is the parenchymatous injection of iodoform in a solution of ether and olive oil, the proportions being seven grammes each of olive oil and ether and one gramme of iodoform. Under this treatment the measurements of the enlarged gland are diminishing rapidly. Gare published the clinical records of twenty-five to thirty cases in which this method was carried out with exceedingly gratifying results. I am speaking, however, only of the hypertrophic form.

DR. WM. J. TAYLOR asked if Dr. Roberts had noticed any especial respiratory difficulty or nervous shock in his operations when working near the sheath of the vessels of the neck or in deep dissection. In two cases coming under his observation which were being operated upon for goitre, everything seemed to be going on well when suddenly during the dissection, in which there had been no hemorrhage of any account, the patient had some respiratory difficulty and rapidly sank, dying in a few hours without any obvious reason.

DR. G. G. DAVIS: There are cases of goitre in which the tumor is not very deeply seated and does not go below the sternum and great vessels, so that it can be easily shelled out, and the hemorrhage is almost *nil.* In other cases the hemorrhage is very marked. I saw one case where the patient died upon the table from it. This happened in the hands of a surgeon of highest skill. Therefore, I think that the seriousness of an operation depends upon the kind of case it is rather than the operation or the surgeon, and accidents might happen in the hands of anyone.

DR. SHOEMAKER: I was present at one of the operations reported, and was very much pleased with the skill of Dr. Roberts in managing the hemorrhage by carefully securing the bleeding vessels. It was one of those thyroids which was not very adherent, and did not desend much below the border of the sternum. The special point of interest to me was in seeing how easily such growths come out when skillfully attacked.

DR. DAVIS: If these were cases of intra-capsular enucleation I would be much interested in hearing about the bleeding and how it was controlled. The substance of the thyroid gland and veins in these cases is so extremely friable that it is almost impossible to control the bleeding by the application of hemostats. They simply tear out; they will not hold at all. Dr. Wolff, of Germany, advocated the intra-capsular enucleation of the enlarged thyroid gland, and the method he recommended was practically to bite away the growth piecemeal, controlling hemorrhage as you go along by means of pressure. It takes exceedingly skillful manipulation to perform a thyroidectomy without hemorrhage by such a method, and most of the other surgeons who discussed his paper preferred the ordinary method of Kocher.

DR. ROBERTS: I did not work inside the capsule, but principally outside. I cut down outside the capsule and shelled out the tumor. The operation was extra-capsular except where the capsule was accidentally cut through during the operation. Of course, the goitres are much less large with us than the goitres seen in Europe. In the greatly enlarged glands the vessels are increased in number and size, as well as the tissue element. I have seen a comparatively large number of goitres at the Woman's Hospital, and I have refused to operate upon a good many of them. It seems to me, however, that where there is dyspnœa or pain from pressure upon the vessels of the nerves by the growth, it is proper to operate. In many cases relief can be obtained by medicine, and if we can succeed in taking the patient's mind off from the goitre the enlarged gland will give less trouble. The cases reported, it seems to me, were proper ones for operation. With regard to Dr. Taylor's question, I have seen profound collapse during an operation upon the neck, although not in case of goitre. There was sudden stoppage of breathing and serious circulatory disturbance. I found that I was making pressure upon the pneumogastric nerve; as soon as I relaxed it the breathing became normal.

